# The aquaporin4-IgG status and how it affects the clinical features and treatment response in NMOSD patients in Egypt

**DOI:** 10.1186/s12883-021-02083-1

**Published:** 2021-02-03

**Authors:** Nirmeen A. Kishk, Walaa Abdelfattah, Nevin M. Shalaby, Hatem S. Shehata, Amr Hassan, Mohamed I. Hegazy, Noha T. Abokrysha, Doaa Abdellatif, Shereen M. Shawky, Sarah S. Abdo, Noha Taha, Amr M. Fouad, Alaa Elmazny, Amany H. Ragab

**Affiliations:** 1 Department of Neurology, Kasr-Alainy Faculty of Medicine, Cairo University, 7 Emtedad al Ikhaa, Maadi, Nile Corniche, Cairo, Egypt; 2 Department of Clinical Pathology, Kasr-Alainy Faculty of Medicine, Cairo University, Cairo, Egypt; 3 Department of Internal Medicine, Kasr-Alainy Faculty of Medicine, Cairo University, Cairo, Egypt

**Keywords:** Neuromyelitis optica spectrum disorder, Auaporin4-IgG status, Treatment response, Disability

## Abstract

**Background:**

In Egypt, the characterization of Neuromyelitis Optica Spectrum Disorder (NMOSD) is lacking.

**Objectives:**

To determine the demographics, clinical features, aquaporin4 antibodies (AQP4-IgG) status, and neuroimaging of Egyptian NMOSD patients.

**Methods:**

Retrospective analysis of 70 NMOSD patients’ records from the MS clinic, Kasr Alainy hospital, between January 2013 and June 2018.

**Results:**

Patients’ mean age was 34.9 ± 9.2 years, and the mean at disease onset was 28.9 ± 10.5 years. Fifty-nine patients had an initial monosymptomatic presentation. AQP4-IgG was measured using either enzyme-linked immunosorbent assay (ELISA) (22 patients) or cell-based assay (CBA) (34 patients). Six and 29 patients had positive results, respectively (*p* < 0.001). 84% had typical NMOSD brain lesions. Longitudinally extensive myelitis was detected in 49 patients, and 9 had either short segments or normal cords. Treatment failure was higher in seropositive patients. Rituximab significantly reduced the annualized relapse rate (ARR) compared to Azathioprine with a percentage reduction of (76.47 ± 13.28) and (10.21 ± 96.07), respectively (*p* = 0.04). Age at disease onset was the only independent predictor for disability (*p* < 0.01).

**Conclusion:**

Treatment failure was higher in seropositive patients. However, there was no difference in clinical or radiological parameters between seropositive and seronegative patients. Patients, who are polysymptomatic or with older age of onset, are predicted to have higher future disability regardless of the AQP4-IgG status.

**Supplementary Information:**

The online version contains supplementary material available at 10.1186/s12883-021-02083-1.

## Background

Neuromyelitis Optica Spectrum Disorder (NMOSD) is a rare inflammatory central nervous system (CNS) disorder of autoimmune etiology [1].

Classic presentations of the disease result from lesions in areas that generally express high levels of aquaporin 4; this refers to the optic nerves, spinal cord, dorsal medulla, brain stem, thalamus, and hypothalamus. More than 50% of patients are expected to lose vision and their ability to walk independently within 5 years of disease onset [2].

Aquaporin 4 immunoglobulins G (AQP4- IgG) present in more than three-quarters of patients constitutes a sensitive and highly specific serum marker of NMOSD, distinguishing the disease from multiple sclerosis [3].

The NMOSD diagnostic criteria published in 2015 are considered one of the core diagnostic characteristics [4].

Unlike multiple sclerosis (MS), the prominent geographical heterogenicity is not yet proven for NMOSD, and it is still unclear whether the disease phenotypes and demographic features vary among different populations.

Significantly few published reports of NMOSD appeared from the middle east [5–7], and to date, much about the disease characteristics in Egypt (one of the most heavily populated countries in the middle east and north Africa region) is still not known.

The current study aimed to elucidate the demographics, clinical features, AQP4- IgG status, neuroimaging, and predictors of disability progression of Egyptian patients with NMOSD.

It is the first study in Egypt to assess the AQP4-IgG using the cell-based assay (CBA) and compare it with the traditional enzyme-linked immunosorbent assay (ELISA).

## Methods

### Study design and participants

Retrospective analysis of patients’ medical records with a working diagnosis of NMO/NMOSD attending the multiple sclerosis clinic, Kasr Alainy hospital, Cairo University, between January 2013 and June 2018 was done.

### Data collection

Detailed clinical, laboratory, and radiological data were extracted by expert neurologists specialized in autoimmune and inflammatory neurological diseases.

Patients who were diagnosed before 2015 had their diagnosis revised according to the 2015 international panel for NMO Diagnosis (IPND) [4]. All patients fit into the revised criteria and were given the diagnosis of NMOSD. A total of 70 patients were enrolled in the study after experienced neurologists reviewed the patients’ data, and patients with suspected alternate diagnoses or missing data were excluded.

Baseline Expanded Disability Status Scale (EDSS) [8] was the one recorded in the patient’s first visit, and the follow-up was the last scored in the outpatient clinic visit within 2 months before data acquisition. The number of relapses for each patient was recorded, and the annualized relapse rate (ARR) was defined as the number of confirmed relapses per year (verified by a neurologist within 7 days after symptom onset) [9]. Treatment failure was considered if new CNS symptoms and signs that lasted longer than 24 h with or without new lesions on gadolinium-enhanced magnetic resonance imaging (MRI) occurred despite the use of immunotherapies [10].

### Imaging

Cranial and spinal MRI with gadolinium was performed when clinically indicated with 1.5 Tesla scanners. MS-like lesions were defined as lesions fulfilling the Barkhofs criteria for multiple sclerosis, and NMOSD typical brain lesions were defined as peri ependymal lesions surrounding the ventricles and the aqueduct, extensive lesions involving corticospinal tracts, hemispheric tumefactive or cloud-like enhancing lesions [11, 12].

### Aquaporin4-IgG testing

Testing for AQP4- IgG was done within 1 month following relapses using either ELISA or CBA by indirect immunofluorescence. Patients tested by CBA were also tested for anti-myelin oligodendrocyte glycoprotein antibodies (anti-MOG-Abs). Only two patients were positive for anti-MOG-Abs and were excluded from the study as well. Cerebrospinal fluid (CSF) analysis for immunoglobulins G (IgG) index and oligoclonal bands (OCB) detection was done in selected subjects who had initially doubtful diagnoses.

### Ethical consideration

The study protocol was approved by the neurology department review board and followed the principles outlined in the declaration of Helsinki. Informed written consent was taken from all participants prior to the enrollment.

### Statistical analysis

SPSS version 18.0 was used for data analysis. Mean ± standard deviation (SD) described quantitative variables and medians with range for data that did not follow normality. The number and percentages described qualitative data, and Chi-square tested proportion independence. For comparing the mean values of two independent groups and more than two independent groups, parametric and non-parametric t-test and one-way ANOVA were used. A paired t-test was used for comparing the means of 2 dependent groups. *P*-values were significant at 0.05. Kruskal Wallis test was used for comparing the percent ARR reduction. Multiple linear regression analysis was used to detect disability predictors, and multivariate analysis was used to describe independent variables that might determine the risk of having an EDSS of > 5.

## Results

### Demographics, clinical and paraclinical characteristics of the study population

#### Clinical characteristics

Our study population included 70 NMOSD patients (58 females and 12 males) with ages ranging from18 to 64 years.

The demographics, clinical and paraclinical characteristics of the study population are outlined in Table 1.

**Table 1:** Demographics, clinical and paraclinical data of the study population

**Age in years** (Mean ± SD)	34.9 ± 9.2
**Age at disease onset in years** *(Mean ± SD)*	28.9 ± 10.5
**Duration to diagnosis in months** *Median (IQR)*	10 (0–180)
**Disease duration in months** *Median (IQR)*	52 (8–216)
**Number of relapses** *(Mean ± SD)*	4.15 ± 2.3
**Baseline EDSS** *(Mean ± SD)*	4.7 ± 2.3
**Last assessed EDSS** *(Mean ± SD)*	4.3 ± 2.3
**Co-morbid autoimmune diseases (***n*)	
Organ-specific	15
Non-organ specific	1
**Family history of immune-mediated diseases** (*n*)	
Systemic Lupus erythematosus	2
Rheumatoid arthritis	3
Multiple sclerosis	1
**MRI cervical spine**	
Normal	1
Lesions < 3 segments	7
Lesions > 3 segments	46
Not done	16
**MRI thoracic spine**	
Normal	3
Lesions < 3 segments	3
Lesions > 3 segments	13
Not done	51
**MRI brain**	
MS-like lesions (periventricular– Juxta cortical)	**5**
Non – specific lesions (migraine-like tiny lesions)	**6**
NMOSD typical lesions	59
**CSF** *(n = 20)*	
Oligoclonal bands	
Positive	9
Negative	11
IgG index	
High	5
Normal	15

*EDSS* Expanded Disability Status Scale, *IQR* Interquartile range, *SD* Standard Deviation, *CSF* cerebrospinal fluid, *IgG* immunoglobulins G, *MRI* magnetic resonance imaging

Twenty-seven (38.6%) patients presented with myelitis, 25 (35.7%) with optic neuritis, 6 (8.6%) with brainstem syndrome, 1 (1.4%) with area postrema syndrome, and 11 (15.7%) had a polysymptomatic presentation.

Motor and sensory symptoms were the most frequently reported by our patients throughout their disease course, followed by sphincteric, then visual complaints Table 2.

**Table 2:** Symptoms frequency during the disease course

Symptom	*(n)*
Limb weakness	54
Sensory disturbances	54
Sphincter troubles	46
Visual loss	39
Fatigue	10
Psychiatric (depressive symptoms)	7
Ataxia	6
Headache	5
Facial paralysis	3
Dysphagia	3
Diplopia/ocular movement abnormalities	2
Dysarthria	2
Cognitive dysfunction	2
Confusion	2
Intractable vomiting	2
Vertigo	1
Seizures	1
Dysgeusia	1
Trigeminal neuralgia	1

#### Aquaporin4-IgG status

Six out of 22 patients (37.5%) tested positive for AQP4-Ab by ELISA compared to 29/34 (85%) by CBA. (*p* < 0.001). Sixteen patients were negative by ELISA, and five were negative by CBA. Fourteen patients presented with typical clinical and imaging features fulfilling the 2015 consensus criteria for NMOSD with negative OCB and IgG index. These 14 patients were not tested for AQP4-IgG because they were on steroids or immunotherapies.

#### Immunotherapy

A total of 32 patients used Azathioprine, rituximab by 12, cyclophosphamide by 11, mitoxantrone by five, and mycophenolate mofetil by two over their disease course. The number of patients who have failed to adequately respond to those drugs when used as first, second, and third drugs is outlined in Table 3.

**Table 3:** Immunotherapy failure when used as first, second, and third therapies

	1st Drug ***N*** = 41 (F/T)	2nd Drug ***N*** = 15 (F/T)	3rd Drug ***N*** = 6 (F/T)
Azathioprine (*n*)	10/22	4/8	2/2
Rituximab(*n*)	3/7	1/2	1/3
Cyclophosphamide(*n*)	4/8	1/2	1/1
Mitoxantrone (*n*)	1/2	3/3	0
Mycophenolate (*n*)	1/2	0	0

*F* Failed, *T* Total

### Comparing patients according to their AQP4-IgG status

#### Demographics and clinical characteristics

The clinical data and demographics of patients with positive and negative AQP4-IgG status are shown in Table 4.

**Table 4:** Demographics and clinical data of patients with positive and negative AQP4-IgG status

	Positive (***n*** = 35)	Negative/Unavailable (***n*** = 35)	***P***-value
Age *(Mean ± SD)*	34.9 ± 9.1	34.8 ± 9.5	0.20
Sex F/M (*n*)	29/6	29/6	1.00
Age at onset *(Mean ± SD)*	30.1 ± 9.3	27.6 ± 11.5	0.30
Number of relapses *(Mean ± SD)*	4.4 ± 2.3	3.8 ± 2.4	0.30
Presentation			
Monosymptomatic (*n*)	30	29	0.50
Polysymptomatic (*n*)	5	6	
Initial monosymptomatic presentation (*n*)			
Brain stem syndrome	5	1	0.30
Transverse myelitis	11	16	
Optic neuritis	13	12	
Area postrema syndrome	1	0	
Baseline EDSS *(Mean ± SD)*	5.1 ± 2.2	4.2 ± 2.3	0.09
Last assessed EDSS *(Mean ± SD)*	5.1 ± 2.3	4.3 ± 2.1	0.08

*AQP4-IgG* aquaporin 4 immunoglobulins G, *EDSS* expanded disability status scale, *F* female, *M* male

#### Radiological findings

MS-like and non-specific MRI brain lesions were found in 6 aquaporin seropositive patients and 5 with a negative status. On the other hand, 29 seropositive and 30 patients with a negative status had NMOSD typical lesions. Table 5 shows MRI cervical and thoracic spine results concerning the aquaporin status.

**Table 5:** The relation of AQP4-IgG status to MRI findings

	Positive (***n*** = 35)	Negative/Unavailable (***n*** = 35)	***P***-value
**MRI cervical spine**			
Normal	0	1	0.50
< 3 segments	2	5	
> 3 segments	23	23	
**MRI thoracic spine**			
Normal	1	2	0.08
< 3 segments	1	2	
> 3 segments	11	2	

*MRI* magnetic resonance imaging

#### Treatment response

Twenty-five patients have undergone plasma exchange for their acute relapses, one out of 15 with a positive aquaporin status, and 2 out of 8 with a negative status failed to respond to the procedure (*p*-value = 0.2). Response to immunotherapies concerning aquaporin status is shown in Table 6.

**Table 6:** AQP4-IgG status and treatment response

	Positive (***n*** = 35)	Negative/Unavailable (***n*** = 35)	***P***-value
**Azathioprine**			
Response	7	9	0.100
Failure	11	5	
**Rituximab**			
Response	5	3	0.600
Failure	3	1	
**Cyclophosphamide**			
Response	0	5	0.020
Failure	5	1	
**Total**			
Response	12	18	0.007
Failure	19	6	

### Comparing patients according to spinal cord lesions length

Forty-nine patients had long extensive transverse myelitis (LETM), and 9 had short segments or normal cords on either the cervical or the thoracic spine MRI scans; their mean ages and ages at disease onset were 34.7 ± 7.8, 35.3 ± .3 (*p* = 0.7), and 28.7 ± 9.2, 29.8 ± 6.5, (*p* = 0.6) respectively. No statistically significant difference was observed when comparing baseline or last assessed EDSS of both groups [5 ± 2.3, 3.9 ± 2, (*p* = 0.2) and 4.4 ± 2.4, 4 ± 2, (*p* = 0.7) respectively].

### Annualized relapse rates with different immunotherapies

The percent reduction of ARR for rituximab and Azathioprine after excluding patients on treatment for less than one-year comparison yielded a statistical significance Table 7.

**Table 7:** Percent reduction in annualized relapse rate with rituximab and Azathioprine

	Rituximab (***n*** = 32)		Azathioprine (***n*** = 12)		***P***-value
	Mean ±	SD	Mean ±	SD	
**Before treatment**					
*No* of relapses	2.14 ±	0.69	2.88 ±	1.67	0.13
Duration of treatment (years)	1 ±	0.57	2.59 ±	2.73	0.03
ARR	2.97 ±	1.8	2.42 ±	1.88	0.53
**After treatment**					
*No* of attacks	0.66 ±	0.51	1.05 ±	0.99	0.23
Duration of treatment (years)	1.05 ±	0.95	1.18 ±	0.94	0.77
ARR	1.4 ±	2.3	1.81 ±	3.02	0.74
**% reduction in ARR**	59.8 ±	42.69	31.96 ±	81.64	0.36
**% reduction in ARR**^a^	76.47 ±	13.28	10.21 ±	96.07	0.04^a^

*ARR* annualized relapse rate, *SD* standard deviation

^a^after exclusion patients on treatment for less than 1 year

### Regression analysis

Multiple linear regression analysis was done to detect disability predictors (Age at onset, disease duration, and the number of relapses). A significant regression equation was found (F (3,62) =4.25, *p* = 0.009) with R2 of 0.17, and the age at onset was the only independent predictor for disability (beta = 0.09, *p* < 0.01) Table 8.

**Table 8:** Multiple regression analysis for disability predictors

Independent variables	Coefficient	Standard error	***P***-value	r_**partial**_	r_**semipartial**_
**Age at onset**	0.09	0.027	0.001*	0.396	0.393
**Disease duration**	0.01	0.008	0.31	0.128	0.117
**Number of relapses**	0.15	0.121	0.22	0.156	0.144

*Significant

Using multivariate analysis, the risk of having an EDSS score of more than 5 in patients with the polysymptomatic presentation was more than three times greater than initial monosymptomatic patients (OR = 3.8; 95% CI = 1.04–13.74; *p* = 0.04) Table 9.

**Table 9:** Multivariate analysis for having an EDSS score of more than 5

Independent variables	Odds ratio	95% CI	***P***-value
AQP4-IgG status (positive vs negative**/**unavailable)	1.6	0.55–4.6	0.39
MRI brain (MS like/non-specific vs NMOSD typical lesions)	3.3	0.7–15.9	0.13
Polysymptomatic presentation	3.8	1.04–13.74	0.04*
MRI spine lesions (short vs long)	2.3	0.71–7.62	0.17
Gender	0.8	0.19–3.75	0.83

*Significant

*MS* multiple sclerosis, *NMOSD* neuromyelitis optica spectrum disorders

## Discussion

As far as we know, this is the first Egyptian study characterizing NMOSD to use CBA for diagnosis and to test for anti-MOG-Abs, besides having a larger number of patients than the previously reported in Egypt.

The mean age of disease onset in this study population was 28.9 ± 10.5 years, which was close to that reported by the only Egyptian study characterizing a cohort of 20 NMOSD patients (27.8 ± 13.71 years) [6], yet it remains lower than those outlined in other studies [7, 13–15].

In the current study, AQP4-IgG status was assessed in 22 patients using ELISA (the only assay available in Egypt before 2017), and in 34 patients using CBA, the latter assay conveys significantly higher sensitivity in detecting AQP4-IgG seropositivity. In this regard, we added the value of utilizing the CBA technique as the most sensitive assay currently available, recommended by the International consensus diagnostic criteria for neuromyelitis optica spectrum disorders and confirmed in previous studies [16, 17].

Despite using the most sensitive technique, AQP4-IgG is not detected in 10–40% of patients diagnosed with NMOSD [18]. Eighty-five percent of the patients tested by CBA in our study were positive for AQP4-IgG. This percentage is higher than in other cohorts from the United States, Malaysia, Thai, Japan, and Korea [19–22].

Statistically significant differences between AQP4-IgG seropositive and seronegative patients regarding the demographics, presenting symptoms, EDSS scores, MRI findings, and CSF results were not observed. It is crucial to note that the different AQP4-IgG testing techniques have likely diluted the differences between the two groups based on ELISA’s presumed false-negative results.

In our cohort, 8.1% of cervical or thoracic MRI lesions in AQP4-IgG positive patients were short segments. This underscores the importance of AQP4-IgG testing in patients having overlapping NMOSD and MS symptoms with short lesions to avoid MS misdiagnosis and, consequently, the use of therapies that might aggravate NMOSD in an account of the non-appreciation of the potential of short transverse myelitis to occur in NMOSD.

Rituximab appeared to provide better disease stabilization than Azathioprine in NMOSD patients in earlier studies [10, 23, 24]. Because of the availability and lower cost of Azathioprine compared to its counterparts, It is more frequently prescribed in our country.

In our patients, treatment with Azathioprine and rituximab resulted in a comparable reduction in ARR. However, after excluding patients on treatment for less than 1 year, rituximab showed significantly higher ARR percent reduction compared to Azathioprine.

Within the AQP4-IgG seropositive patients, the number of patients who failed immunotherapies was significantly higher than those with a negative status in our cohort. A study in 2018 described similar findings in their study that seropositive patients utilized significantly higher immunotherapies [25].

Disability determinants of our cohort revealed that age at onset was the only independent predictor by multivariant regression analysis; this is consistent with a study’s findings in 2012, which concluded that older age at disease onset was predictive of motor disability [26].

Some caveats need to be noted in our study, including the retrospective nature of the analysis, which could have introduced bias in data collection, the heterogenicity of AQP4-IgG testing techniques, and none of the patients tested using ELISA were tested for anti-MOG-Abs. The strengths included the relatively large number of patients enrolled, the use of CBA for diagnosis (first Egyptian NMO study to use the technique), and the anti-MOG-Ab testing in most patients.

## Conclusion

Our findings contribute in a way to the emerging picture of NMOSD in the region. Treatment failure was higher in seropositive patients. However, there was no difference in clinical or radiological parameters between seropositive and seronegative patients. Patients, who are polysymptomatic or with older age of onset, are predicted to have higher future disability regardless of the AQP4-IgG status. Rituximab treatment resulted in a significant percentage reduction in ARR compared to Azathioprine.

## Supplementary Information

**Additional file 1.**

## Data Availability

The datasets used and/or analyzed during the current study are available in the supplementary document.

## Abbreviations

NMOSD: Neuromyelitis Optica Spectrum Disorder
CNS: Central nervous system
AQP4-IgG: Aquaporin 4 immunoglobulins G
MS: Multiple sclerosis
ELISA: Enzyme-linked immunosorbent assay
CBA: Cell-based assay
IPND: International Panel for NMO Diagnosis
EDSS: Expanded Disability Status Scale
ARR: Annualized relapse rate
MRI: Magnetic resonance imaging
Anti-MOG-Abs: Anti-myelin oligodendrocyte glycoprotein antibodies
CSF: Cerebrospinal fluid
IgG: Immunoglobulins G
OCB: Oligoclonal bands
SD: Standard deviation
IQR: Interquartile range
